# Two *FgLEU2* Genes with Different Roles in Leucine Biosynthesis and Infection-Related Morphogenesis in *Fusarium graminearum*

**DOI:** 10.1371/journal.pone.0165927

**Published:** 2016-11-11

**Authors:** Xin Liu, Qi Han, Jian Wang, Xin Wang, Jianhong Xu, Jianrong Shi

**Affiliations:** 1 Institute of Food Quality and Safety, Jiangsu Academy of Agricultural Sciences, Nanjing, Jiangsu, China; 2 Key Lab of Food Quality and Safety of Jiangsu Province-State Key Laboratory Breeding Base, Nanjing, Jiangsu, China; 3 Key Laboratory of Control Technology and Standard for Agro-product Safety and Quality, Ministry of Agriculture, Nanjing, Jiangsu, China; 4 Key Laboratory of Agro-product Safety Risk Evaluation (Nanjing), Ministry of Agriculture, Nanjing, Jiangsu, China; 5 Collaborative Innovation Center for Modern Grain Circulation and Safety, Nanjing, Jiangsu, China; Seoul National University, REPUBLIC OF KOREA

## Abstract

3-isopropylmalate dehydrogenase (IPMD) encoded by *LEU2* is a key enzyme in leucine (Leu) biosynthetic pathway. Analysis of the genome sequence of *Fusarium graminearum* revealed two paralogous *LEU2* genes (designated as *FgLEU2A* and *FgLEU2B*) in this fungus and the deduced amino acid sequences of FgLeu2A and FgLeu2B share 45% identity. Targeted disruption of individual *FgLEU2A/B* gene in *F*. *graminearum* assigned a more crucial role of FgLeu2A in Leu biosynthesis as disruption of *FgLEU2A* resulted in mutant (ΔFgLeu2A-10) that was Leu-auxotrophic and could not grow in minimal medium limited for amino acids, whereas *FgLEU2B* deletion mutant ΔFgLeu2B-2 was morphologically indistinguishable from the wild type strain PH-1. The growth defects of ΔFgLeu2A-10 could be overcome by exogenous addition of Leu at 0.25 mM. Double deletion of *FgLEU2A* and *FgLEU2B* (ΔFgLeu2AB-8) caused a more severe Leu-auxotrophic phenotype as the concentration of Leu exogenously added to medium to rescue the growth defect of ΔFgLeu2AB-8 should be raised to 1.25 mM, indicating a less important but nonnegligible role of FgLeu2B in Leu biosynthesis. Disturb of Leu biosynthesis caused by *FgLEU2A* deletion leads to slower growth rate, reduced aerial hyphal formation and red pigmentation on PDA plates and completely blocked conidial production and germination. All of the defects above could be overcome by Leu addition or complementation of the full-length *FgLEU2A* gene. ΔFgLeu2A-10 also showed significantly increased sensitivity to osmotic and oxidative stresses. Pathogenicity assay results showed that virulence of mutants lacking *FgLEU2A* were dramatically impaired on wheat heads and non-host cherry tomatoes. Additionally, a low level of deoxynivalenol (DON) production of ΔFgLeu2A-10 and ΔFgLeu2AB-8 in wheat kernels was also detected. Taken together, results of this study indicated a crucial role of FgLeu2A and a less important role of FgLeu2B in Leu biosynthesis and fungal infection-related morphogenesis in *F*. *graminearum* and FgLeu2A may serve as a potential target for novel antifungal development.

## Introduction

Branched-chain amino acids (BCAAs) including leucine (Leu), valine (Val) and isoleucine (Ile) can be synthesized in bacteria, fungi and high plants, but not mammals. Mammals must acquire BCAAs from their diet and the absence of BCAAs biosynthetic pathway in mammals make the enzymes involved attractive targets for the development of herbicides and anti-microorganisms [1,2]. Recent studies have revealed that BCAAs, especially Leu, not only serve as fundamental substrates for protein synthesis but also have unique ability to initiate signal transduction pathways that modulate translation initiation [3,4].

Leu is synthesized as a branch that leads from 2-ketoisovalerate (2-KIV), the intermediate precursor of Val, to Leu and its biosynthesis contains four enzymatic reactions catalyzed by isopropylmalate synthase (IPMS, encoded by *LEU4*), isopropylmalate isomerase (IPMI, encoded by *LEU1*), isopropylmalate dehydrogenase (IPMD, encoded by *LEU2*) and branched-chain aminotransferase (BCAT, encoded by *BCA1*/*2*) [1]. The third Leu biosynthetic specific enzyme IPMD catalyzes the conversion of 3-isopropylmalate (3-IPPM) to 2-ketoisocaproate (2-KIC) and its encoding gene *LEU2* is one of the best-studied BCAA biosynthetic genes because of its wide use as selected marker for gene-transformation experiments in *Saccharomyces cerevisiae* and several other yeast species [5,6].

In *S*.*cerevisiae*, functional analysis showed that disruption of *LEU2* resulted in Leu-auxotroph and decreased resistance to acetic acid and to very high levels of ethanol stress of the mutants [7]. Very few documents characterized functions of *LEU2* in filamentous fungi except for a relatively ancient and primary research in *Aspergillus niger* showed that this fungus elaborates two isoenzymes of IPMD and these two encoding genes were differentially expressed [8]. However, the detailed function of these two paralogous *LEU2* genes in Leu biosynthesis or other cellular processes was not investigated in *A*. *niger* or other filamentous fungi.

Fusarium head blight (FHB) caused by the pathogenic ascomycete fungus *Fusarium graminearum* (teleomorph *Gibberella zeae*) is one of the most important diseases in cereal crops including wheat and barley worldwide [9–11]. FHB epidemics would not only cause huge yield losses but also bring health threaten and food safety concern to humans and animals as the fungus produces mycotoxins such as deoxynivalenol (DON) during infection [9,12]. Disease control of FHB and mycotoxin contaminations remains challenging due to the lack of resistant wheat cultivars to FHB and limited effective fungicides against *F*. *graminaerum*. Therefore, it is extremely urgent to develop novel, less-toxic and effective fungicides for the management of FHB. Enzymes involved in certain amino acids biosynthesis (e.g., BCAAs) have been regarded as attractive fungicide targets due to their absence in mammals and the important biological functions of the genes encoding for these enzymes in yeasts and filamentous fungi [13–21].

In this study, we found two paralogous *LEU2* genes in the genome of *F*. *graminearum*, using targeted-gene disruption strategy we have constructed single and double deletion mutants of *FgLEU2A/B* gene in *F*. *graminearum* and investigated the different roles of each gene in Leu biosynthesis and in other important cellular processes. Our results are in consistence with previous findings that BCAAs biosynthesis is important event and FgLeu2A plays more important roles in Leu biosynthesis and infection-related morphogenesis and can be regarded as potential target for novel fungicide development.

## Materials and Methods

### Strains, growth conditions and phenotype assays

The *Fusarium graminearum* strain PH-1 (a Michigan field isolate, USA origin, FGSC 9075, NRRL31084 [22]) was used as the parental wild-type. To assess the mycelial growth and colony characteristics, the wild-type strain PH-1 and mutants were cultured on different medium including potato dextrose agar (PDA), yeast extract peptone glucose agar (YEPD), fructose gelatin agar (FGA) or minimal medium (MM) plates supplemented with or without different amino acids and incubated at 25°C. Colony diameter was measured every 12 h. Fungal biomass analysis was conducted by collecting mycelia from 2-days-old liquid culture, and then washed with sterilized water, dried in 65°C bake oven and determined by mycelial dry weight. Experiments were performed triplicate. *Escherichia coli* DH5α was cultured at 37°C and used for bacterial transformations. For conidiation assay, five 5-mm mycelial plugs of the wild-type strain and mutants taken from the edge of a 3-day-old colony were inoculated in a 50-ml triangular flask containing 20 ml of MBL (40 g mung beans boiled in 1 L water for 20 min, and then filtered through cheesecloth) for conidiation. The flasks were incubated at 25°C for 4 days in a shaker (180 rpm). The amount of conidia produced by the *F*. *graminearum* strains in MBL was counted using a hemacytometer. Additionally, conidial germination was carried out by re-suspending the conidia of the wild-type strain and mutants in 2% sterilized sucrose solutions supplemented with or without different amino acids at 25°C for 4 h and 6 h, conidial germination was examined under a Nikon ECLIPSE E100 microscope (Nikon Co., Tokyo, Japan). Each experiment was carried out with three replicates.

### Sequence analysis of *FgLEU2A*/*B* genes in *F*. *graminearum*

Using the amino acid sequence of *S*. *cerevisiae* IPMD Leu2 (Saccharomyces database accession numberYCL018W) as query, two paralogous FgLeu2A (FGSG_06675.3) and FgLeu2B (FGSG_10671.3) were identified through a homology search in the *F*. *graminearum* genome database (available at http://fungidb.org/fungidb/). Transcript of the *F*. *graminearum FgLEU2A* or *FgLEU2B* gene was detected by using reverse transcription PCR (RT-PCR) with primer pairs Leu2A-all-F + Leu2A-all-R or Leu2B-all-F + Leu2B-all-R (S1 Table), using cDNA and genomic DNA as templates, respectively. RNA was extracted from mycelia of the wild-type PH-1 using a TaKaRa RNAiso Reagent (TaKaRa Biotech. Co., Dalian, China), and used for reverse transcription with the primer oligo(dT)_18_ using a PrimeScript^™^ RT reagent kit (TaKaRa Biotech. Co.,Dalian, China). PCR amplifications were conducted using the following parameters: initial denaturation at 95°C for 3min, followed by 30 cycles of denaturation at 94°C for 45 s, annealing at 53°C for 45s, extension at 72°C for 1.5 min, and final extension at 72°C for 10 min. PCR products were detected on 1% agarose gels in Tris-acetate (TAE) buffer and photographed after staining with ethidium bromide. The resultant PCR product was purified, cloned and sequenced.

### Construction of *FgLEU2A/B* deletion mutants

*FgLEU2A* deletion vector pBS-leu2A-Del was constructed by inserting two flanking sequences of *FgLEU2A* gene into the left and right sides of the hygromycin resistance gene *HPH* in the pBS-HPH1 vector [23]. First, a 651-bp upstream flanking sequence fragment of *FgLEU2A* was amplified by using primer pair A1 + A2 (S1 Table) from the genomic DNA of PH-1, and was inserted into *Kpn*I-*Xho*I sites of the pBS-HPH1 vector to generate pBS-leu2A-up. Subsequently, a 941-bp downstream flanking sequence fragment of *FgLEU2A* amplified from PH-1 genomic DNA using the primers A3 + A4 (S1 Table) was inserted into *Bam*HI-*Sac*I sites of the pBS-leu2A-up vector to generate pBS-leu2A-Del. Similar to pBS-leu2A-Del, we constructed *FgLEU2B* deletion vector pBS-leu2B-Del (see S2A and S3A Figs). *FgLEU2A/B* double deletion vector pBS-leu2AB-Del was constructed by modifying pBS-leu2A-Del. The G418 sulfate resistance gene *NEO* amplified by using primer pair Neo-F + Neo-R from plasmid pBS-RP-Red-A8-NEO (S1 Table) was introduced to the *Xho*I-*Bam*HI sites of pBS-leu2A-Del. Finally, the 3095-, 2708- or 3455-bp fragments containing leu2A/B-upstream-HPH(NEO)-leu2A/B-downstream cassette was obtained by PCR amplification with primer pair A1 + A4 or B1+ B4 using diluted plasmid pBS-leu2A/leu2B/leu2AB-Del as templates. The resultant PCR product was purified and stored at -20°C for protoplast transformation.

The PEG-mediated protoplast fungal transformation was performed as described previously [24]. PDA medium supplemented with hygromycin (100 mg/L) was used to select the resistant transformants. After single spore isolation and PCR identification, transformants were kept at 4°C for further experiments.

### Complementation of *FgLEU2A* deletion mutant

To confirm that the phenotype of *FgLEU2A* deletion mutant is caused by disruption of the gene, the *FgLEU2A* deletion mutant ΔFgLeu2A-10 (hereafter named ΔFgLeu2A-10) was complemented with the full-length *FgLEU2A* containing its promoter and terminator regions. The complemented plasmid pCA-leu2A-C was constructed using the backbone of pCAMBIA1300. First, G418 sulfate resistance cassette was amplified from plasmid pBS-RP-Red-A8-NEO with primers Neo-F + Neo-R (S1 Table), and cloned into the *Xho*I-*Kpn*I site of pCAMBIA1300 to create plasmid pCA-NEO. Then, a 3437-bp of full-length *FgLEU2A* gene including 997-bp upstream and 1313-bp terminator region was amplified using primers leu2A-com-F + leu2A-com-R (S1 Table) from the genomic DNA of wild-type. This fragment was cloned into the *Bam*HI-*Xba*I sites of pCA-NEO to generate the complement plasmid pCA-leu2A-C. Finally, the 4440-bp fragment containing neo- full-length leu2A cassette was obtained by PCR amplification with primer pair Neo-F + leu2A-com-R using diluted plasmid of pCA-leu2A-C as templates (see s S2A Fig). PCR products were separated on 1% agarose gels in TAE buffer and photographed after staining with ethidium bromide. The resultant PCR product was purified and stored at -20°C for protoplast transformation. Transformation of the protoplasts of ΔFgLeu2A-10 with the full-length *FgLEU2A* was conducted as described above except that G418 sulfate (100 mg/L) was used as a selection agent.

### Amino acids or intermediate products assays

Exogenous amino acids or intermediate products were supplemented to FGA at different concentrations as indicated in figures. Five 5-mm mycelial plugs of the wild-type strain and mutants were taken from the edge of a 3-day-old colony and incubated in the plates described above. Experiments were performed in triplicate and plates were photographed two days after inoculation.

### Plant infection assays

Conidia were harvested from 4-day-old cultures of the wild-type strain and each mutant, then washed with sterilized water and re-suspended in 0.01% (vol/vol) Tween 20 solution and adjusted to 10^5^ conidia/ml. Plant infection assays were performed using single floret injection method as previously described [25]. Briefly, 10 μl of conidial spores (10^5^conidia/ml) was injected into a single floret in the central section spikelet of single flowering wheat heads of susceptible cultivar Jimai19at early anthesis, 10 spikes were used for each strain. Infected spikelets in each inoculated wheat head were recorded 10 days after inoculation. The experiment was repeated for three times.

To examine the ability to colonize cherry tomato, a 10 μl aliquot of conidial suspension (10^5^conidia/ml) was injected into the wounded tomato after surface sterilization. There are five replicates for each strain. Inoculated tomatoes were incubated at 25°C and 100% humidity with 12 h of daylight, and were photographed 3 days after inoculation. The experiment was repeated for three times.

### Determination of sensitivity of the *FgLEU2A/B* mutants to cell stresses

Mycelial growth tests were performed on FGA plates amended with 1.25mMLeu and supplemented with the following chemicals: osmotic stress NaCl, D-sorbitol, KCl and glycerol at 1.2 M; oxidative stress H_2_O_2_ at 12 mM; cell wall inhibitors congo red at 0.4 mg/ml and caffeine at 5 mM; 0.005% cell membrane damager SDS. The percentage of inhibition of mycelia radial growth (PIMG) was calculated using the formula PIMG = [(C-N)/(C-5)] * 100, where C is the colony diameter of the non-treatment, and N is that of the chemical treatment. Each experiment was carried out with three replicates and the experiments were repeated twice.

### DON production analysis

Diseased wheat kernels were harvested from inoculated spikelets 20 days after inoculation and used for DON production analysis using a previously described protocol [26, 27].The amount of DON in each sample was determined using a high-performance liquid chromatography-mass spectrometer/mass spectrometer (HPLC-MS/MS) system (Agilent1100-6410, Agilent Technologies, Palo Alto, Calif.). Mass spectrometric parameters were performed using a previously described detection method [28].

Additionally, the amount of *F*. *graminearum* ergosterol in each sample was also determined. Total ergosterol was extracted from infected wheat powder by using a previously published protocol [29]. Ergosterol concentrations were quantified with a high-performance liquid chromatography (HPLC) system, Waters 1525–2489. Ergosterol was separated at room temperature on a hypersil BDSC18 250 nm×4.6 nm, 5μm analytical column using 100% methanol (chromatography pure) as mobile phase. The detection wavelength was 282 nm. The identification of ergosterol was based on retention time and co-chromatography of commercial standard of ergosterol (Sigma-Aldrich, St. Louis, MO, USA).

### Standard molecular methods

Fungal genomic DNA was extracted using a previously published protocol [30]. Plasmid DNA was isolated using a plasmid miniprep purification kit (BioDev Co., Beijing, China). Southern blot hybridization analysis of *FgLEU2A/B* genes in the wild-type and mutants was performed using probes as indicated in S2A and S3A Figs. Probe was labeled with digoxigenin (DIG) using a high prime DNA labeling and detection starter kit II according to the manufacturer’s instructions (Roche Diagnostics, Mannheim, Germany).

### Data analysis

Statistical comparisons were made between the wild type parent and the mutants by one-way ANOVAs conducted using the software package SPSS (version 13 for Windows, 2004). And for the relative gene expression data, one-sample *t*-test was conducted to test the difference between different time points or different strains using SPSS.

## Results

### Two *FgLEU2* orthologs in *F*. *graminearum*

Unlike the model fungi *S*.*cerevisea*, *F*. *graminearum* has two putative *LEU2* genes that were assigned as *FgLEU2A* (FungiDB accession number: FGSG_00675.3) and *FgLEU2B* (FGSG_10671.3) in this study. FgLeu2A and FgLeu2B share 45% identity in amino acid level.

*FgLEU2A* is 1313-bp in length and encodes 367 amino acids. *FgLEU2A* has four predicted introns and are 53-bp in length located between the 52^th^ and 104^th^ nucleotide, 55-bp in length located between the 126^th^ and 180^th^ nucleotide, 50-bp in length located between the 253^h^ and 302^th^ nucleotide, and 54-bp in length located between the 1024^th^ and 1077^th^ nucleotide, respectively. *FgLEU2B* is 1086-bp in length and encodes 362 amino acids. *FgLEU2B* has no predicted intron. Sequencing of the 1101-bp and 1086-bp cDNA of *FgLEU2A* and *FgLEU2B*, respectively, verified the existence of the predicted introns.

Phylogenetic analysis was performed with the neighbor-joining method using MEGA 4.1 software [31] and revealed two separated branches of Leu2 (Group1 and Group 2) in fungi. Yeast species contain a single Leu2 clustered in Group 1 in their genome while in the filamentous fungal species; some contain single Leu2 while others possess two Leu2 orthologs in their genome. As shown in S1 Fig, both the *Fusarium* and *Aspergillus* species contains two Leu2 orthologs clustered in both Group 1 and Group 2, however, other fungal species including *Magnaporthe oryzea*, *Ustilago maydis* and *Sporisorium reilianum* also contain two Leu2 orthologs in their genome with both Leu2 clustered together in the Group 1 branch with yeast and *Neurospora* species. FgLeu2a is more closely to its *S*. *cerevisiae* and *N*. *crassa* homologues than to FgLeu2b.

### Expression pattern of *FgLEU2A* and *FgLEU2B* in different cultures in *F*. *graminearum*

**Fig 1:**
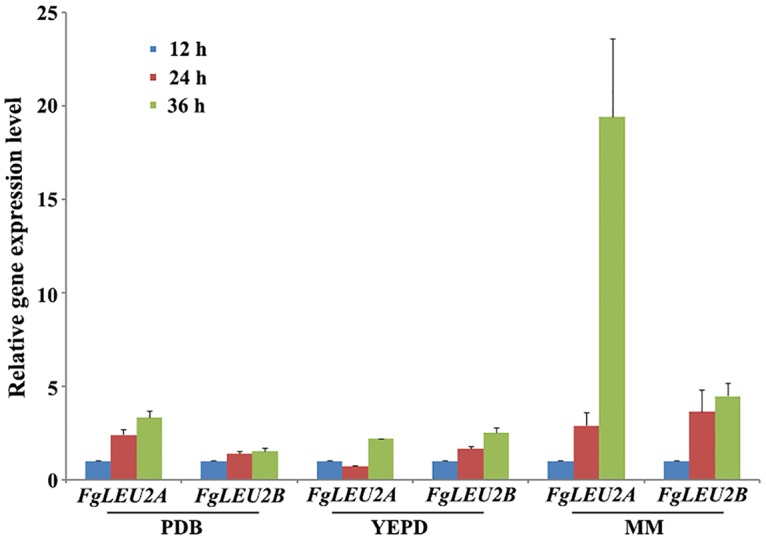
Expression levels of *FgLEU2A* and *FgLEU2B* determined by qRT-PCR in *F*. *graminearum*. Relative expression levels of *FgLEU2A* and *FgLEU2B* in germlings and vegetative hyphae cultured in different medium. The expression levels of each gene in 12 h germlings was arbitrarily set to 1.Differences between the relative expressions (fold changes) of *FgLEU2A/B* at different time points were compared by one sample *t*-test.

Real-time PCR assays showed that when cultured in low-nutrient medium MM containing no amino acids, both *FgLEU2A* and *FgLEU2B* genes were significantly (*p*<0.01) up-regulated in vegetative hyphae as compared to 12 h germlings, the relative expression level of *FgLEU2A* gene raised to 2.90- and 19.42-fold and *FgLEU2B* gene raised to 3.65- and 4.48-fold at 24 h and 36 h, respectively (Fig 1).

### Generations of mutants of *FgLEU2A* and *FgLEU2B* in *F*. *graminearum*

Single deletion mutants of *FgLEU2A* were generated by transforming the 3095-bp fragment containing leu2A-upstream-HPH-leu2A-downstream to the protoplasts of the wild-type, resulting in more than 20primary hygromycin-resistant transformants. Complementation of *FgLEU2A* mutants was conducted by transforming the 4553-bp fragment containing NEO-leu2A-upstream-leu2A-downstream to the protoplasts of the *FgLEU2A* deletion mutants, resulting in more than 25primary G418 sulfate -resistant transformants. First, PCR analysis with primers A5 + A6 (S1 Table) was used for detection of *FgLEU2A* mutants. This primer pair amplified a 2167- and 1785-bp fragment from wild type strain PH-1 and the *FgLEU2A* deletion mutant, respectively, and both from the *FgLEU2A* complementation mutant. The deletion mutant ΔFgLeu2A-10 and the complementation mutant ΔFgLeu2A-8C were chosen for further analysis. Subsequently, reverse transcription PCR with the primer pair leu2A-RT-F + leu2A-RT-R amplified an expected 246-bp fragment from the wild-type progenitor PH-1 and ΔFgLeu2A-8C, but not from the *FgLEU2A* deletion mutant ΔFgLeu2A-10 (S2B Fig). PCR results were further verified by Southern blotting analysis. The genomic DNA of PH-1, ΔFgLeu2A-10 and ΔFgLeu2A-8Cdigested by *Nco* I were blotted and hybridized with a DIG-labeled *FgLEU2A*downstream fragment (S2A Fig). The wild type strain had an expected hybridizing band of 5775-bp, while this band was replaced by a 4279-bp fragment in ΔFgLeu2A-10 (S2C Fig). Additionally, the southern hybridization pattern of ΔFgLeu2A-8C confirmed that a single copy of the full-length *FgLEU2A* was re-introduced into the genome of FgLeu2A-10 (S2C Fig).

Single deletion mutants of *FgLEU2B* were generated by transforming the 3455-bp fragment containing leu2B-upstream-HPH-leu2B-downstream to the protoplasts of the wild-type, resulting in more than 30 primary hygromycin-resistant transformants. First, PCR analysis with primers B5 + B6 (S1 Table) was used for detection of *FgLEU2B* deletion mutants. This primer pair amplified a 1438- and 1778-bp fragment from wild type strain PH-1and *FgLEU2B* deletion mutant, respectively. The mutant ΔFgLeu2B-2 showing the deletion mutant size fragment was chosen for further analysis. Subsequently, reverse transcription PCR with the primer pair leu2B-RT-F + leu2B-RT-R amplified an expected 220-bp fragment from the wild-type progenitor PH-1, but not from the *FgLEU2B* deletion mutantΔFgLeu2B-2 (S3B Fig). PCR results were further verified by Southern blotting analysis. The genomic DNA of PH-1 and ΔFgLeu2B-2 digested by *Nco* I were blotted and hybridized with a DIG-labeled *FgLEU2B* downstream fragment (S3A Fig). The wild type strain had an expected hybridizing band of 4664-bp, while this band was replaced by a 3621-bp fragment in ΔFgLeu2B-2 (S3C Fig).

Double deletion mutants of *FgLEU2A* and *FgLEU2B* were generated by transforming the 2708-bp fragment containing leu2A-upstream-NEO-leu2A-downstream to the protoplasts of ΔFgLeu2B-2, resulting in more than 15 primary G418 sulfate-resistant transformants. First, PCR analysis with primers A5 + A6 (S1 Table) was used for detection of *FgLEU2AB* double deletion mutants. This primer pair amplified a 2167- and 1398-bp fragment from wild type strain PH-1and *FgLEU2AB* deletion mutant, respectively. The double deletion mutant ΔFgLeu2AB-8 showing the deletion mutant size fragment was chosen for further analysis. Subsequently, reverse transcription PCR with the primer pair leu2A-RT-F + leu2A -RT-R amplified an expected 246-bp fragment from the wild-type progenitor PH-1, but not from ΔFgLeu2AB-8 (S2B Fig). PCR results were further verified by Southern blotting analysis. The genomic DNA of PH-1 and ΔFgLeu2AB-8 digested by *Nco* I were blotted and hybridized with a DIG-labeled *FgLEU2A*downstream fragment (S2A Fig). The wild type strain had an expected hybridizing band of 5775-bp, while this band was replaced by a 3776-bp fragment in ΔFgLeu2AB-8 (S2C Fig).

### Disruption of *FgLEU2A* leads to defects in mycelial and conidial morphogenesis in *F*. *graminearum*

**Fig 2:**
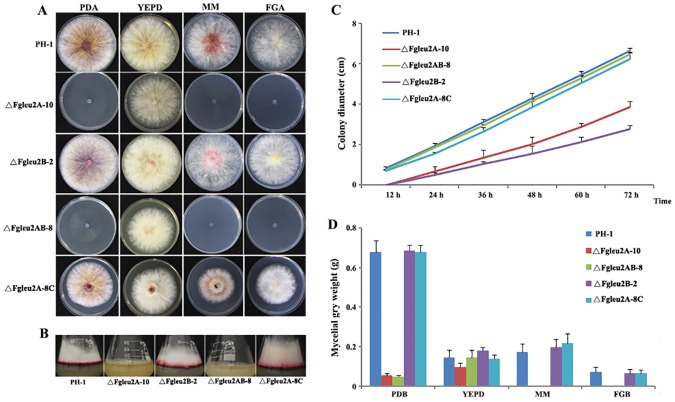
*FgLEU2A* plays essential roles in mycelial growth in *F*. *graminearum*. **A** Colony morphology of the *FgLEU2A* gene deletion mutant (ΔFgLeu2A-10), the *FgLEU2B* gene deletion mutant (ΔFgLeu2B-2) and the *FgLEU2A*/*B* double deletion mutant (ΔFgLeu2AB-8) compared with wild type strain PH-1 and the *FgLEU2A* gene complemented transformant (ΔFgLeu2A-8C) on PDA, MM, FGA or YEPD medium after 3 days of incubation at 25°C. **B** Reduced aerial hyphae of ΔFgLeu2A-10 and ΔFgLeu2AB-8 compared with wild type strain PH-1, ΔFgLeu2B-21 and ΔFgLeu2A-8Con PDA medium after 3 days of incubation at 25°C. **C** Reduced mycelial growth rate of ΔFgLeu2A-10 and ΔFgLeu2AB-8 compared with the wild type strain PH-1, ΔFgLeu2B-2 and ΔFgLeu2A-8C on PDA medium. **D** Fungal biomass of ΔFgLeu2A-10 and ΔFgLeu2AB-8 compared with the wild type strain PH-1, ΔFgLeu2B-2 and ΔFgLeu2A-8C in liquid PDB, YEPD, FGB, and MM after 2 days of incubation at 25°C.

Mycelial morphogenesis was affected by disruption of *FgLEU2A* but not *FgLEU2B*. Mutants lacking *FgLEU2A* (ΔFgLeu2A-10 and ΔFgLeu2AB-8) could not survive in MM and FGA medium and showed a slower mycelial growth rate and reduced aerial hyphal formation on PDA plates as compared to wild type strain PH-1 andΔFgLeu2A-8C (Fig 2A–2C), although microscopy examination revealed no visible differences in hyphal structure between both deletion mutants and the wild type strain. Additionally, when cultured in liquid medium, the mycelial dry mass of ΔFgLeu2A-10 and ΔFgLeu2AB-8 was significantly (*p*<0.05) reduced in PDB and remained undetectable in FGB or MM (Fig 2D).

**Fig 3:**
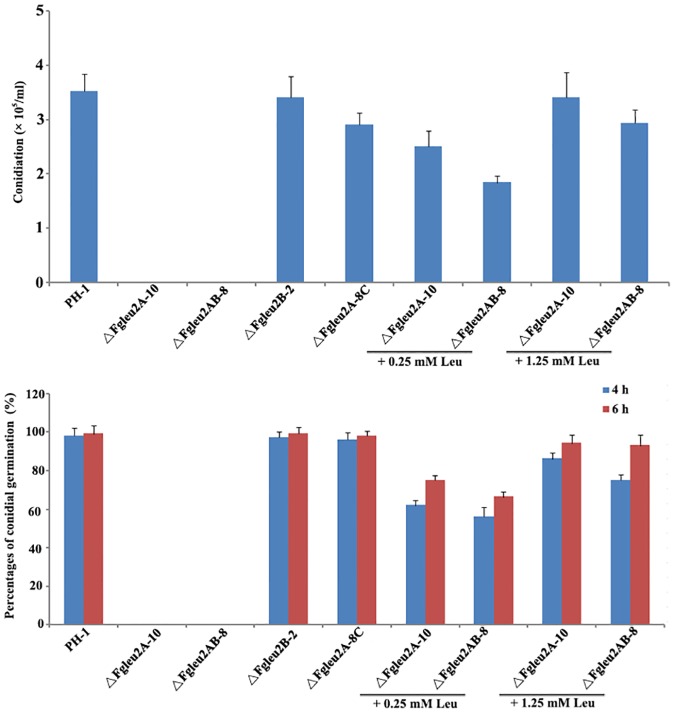
*FgLEU2A* is involved in conidial formation and germination in *F*. *graminearum*. **A** Comparison of conidiation among PH-1, ΔFgLeu2A-10, ΔFgLeu2B-2, ΔFgLeu2AB-8 and ΔFgLeu2A-8C in MBL amended with or without Leu. Line bars in each column denote standard errors of three repeated experiments. **B** Conidia of PH-1, ΔFgLeu2A-10, ΔFgLeu2B-2, ΔFgLeu2AB-8 and ΔFgLeu2A-8C harvested from MBL supplemented with 1.25 mM Leu were re-suspended in 2% sucrose solution and cultured at 25°C for 4h and 6h with or without addition of Leu. Germination rate of each strain was calculated and line bars in each column denote standard errors of three repeated experiments.

To further evaluate the effects of *FgLEU2A* and *FgLEU2B* on conidial morphogenesis, the wild type PH-1 and mutants were cultured in MBL and the amount of conidia was compared. Results showed that when cultured in MBL conidial formation was completely blocked in *FgLEU2A* deleted mutants and the defects in conidial formation could be partially rescued by exogenous supplementation of Leu at 0.25 mM and completely restored at 1.25 mM (Fig 3A). Conidial germination assay was conducted using a modified protocol, conidia were collected from MBL culture amended with 1.25 mM Leu, washed with sterilized water and incubated in 2% sucrose solutions with or without Leu. Very few conidia of ΔFgLeu2A-10 and ΔFgLeu2AB-8 could germinate in 2% sucrose solutions and germination rates of ΔFgLeu2A-10 and ΔFgLeu2AB-8 raised to the wild-type level by addition of 1.25 mM Leu (Fig 3B).

Deletion of *FgLEU2B* caused no visible differences in mycelial and conidial morphogenesis as compared to the wild-type strain PH-1. Complementation of ΔFgLeu2A-10 with full-length *FgLEU2A* gene rescued all the defects of the deletion mutant.

### Deletion of *FgLEU2A* results in reduced red pigmentation and down-regulation of genes involved in red pigment formation in *F*. *graminearum*

**Fig 4:**
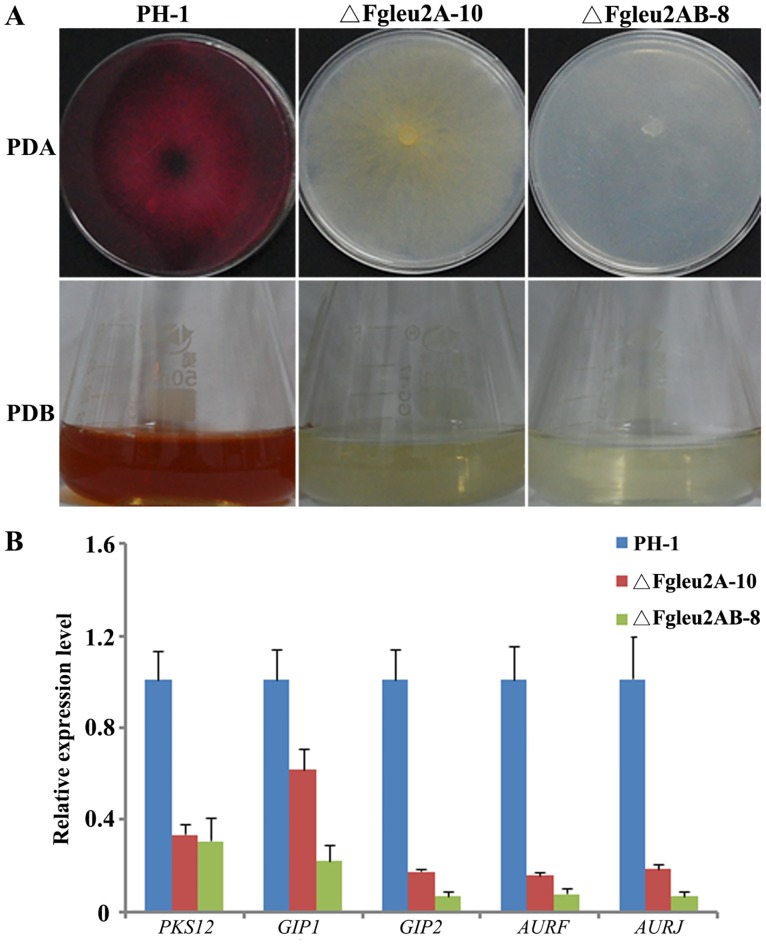
*FgLEU2A* plays important roles in red pigment aurofusarin formation in *F*. *graminearum*. **A** Red pigment aurofusarin formation of the wild-type PH-1, ΔFgLeu2A-10 and ΔFgLeu2AB-8 cultured on PDA or PDB at 25°C for 3 days. **B** The relative expression of each red pigment aurofusarin biosynthetic gene in the deletion mutant ΔFgLeu2A-10 and ΔFgLeu2AB-8 was relative to amount of cDNA of each corresponding gene in the wild type progenitor PH-1. There were significant differences between the relative expression (fold changes) of each red pigment biosynthetic among the wild type PH-1 and ΔFgLeu2A-10 or ΔFgLeu2AB-8 (*p*<0.05).

Apart from the defects in mycelial and conidial morphogenesis, disruption of *FgLEU2A* also leads to reduced accumulation of red pigment (Figs 2B and 4A). Further analysis of the expression level of genes involved in red pigmentation revealed that all five genes (*PKS12*, *GIP1*, *GIP2*, *AURF*, *AURJ*) were significantly down-regulated (*p*<0.05) in ΔFgLeu2A-10 and ΔFgLeu2AB-8 as compared to the wild-type strain PH-1(Fig 4B). We also noticed that the double deletion mutant FgLeu2AB-8 showed a more severe defect in red pigmentation (Fig 4A) and a lower relative expression level of the five red pigment biosynthetic genes than the single deleted mutant ΔFgLeu2A-10 (Fig 4B).

### FgLeu2A plays a more crucial role in Leu biosynthesis in *F*. *graminearum*

**Fig 5:**
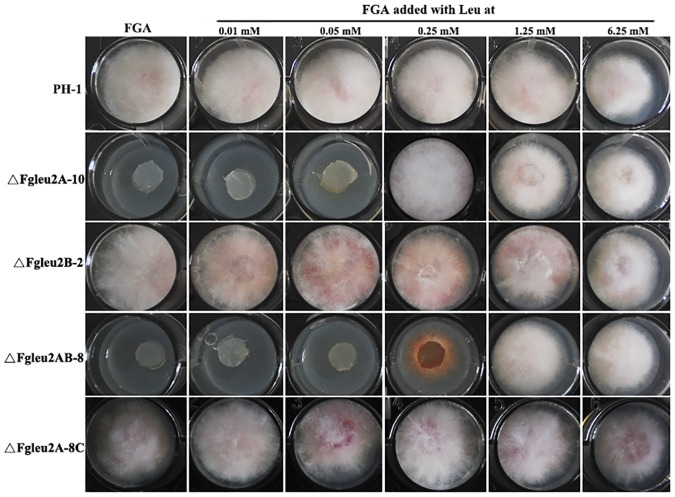
Different roles of *FgLEU2A* and *FgLEU2B* in Leu biosynthesis in *F*. *graminearum*. Mycelial morphology of the wild-type PH-1, ΔFgLeu2A-10, ΔFgLeu2B-2, ΔFgLeu2AB-8 and ΔFgLeu2A-8C cultured on FGA medium amended with Leu at different concentrations indicated in the figure at 25°C for 2 days.

As shown in Fig 1A, deletion of *FgLEU2A* resulted in amino acid auxotroph in *F*. *graminearum*. ΔFgLeu2A-10 and ΔFgLeu2AB-8 could not survive in MM and FGA lacking amino acids. The auxotroph of ΔFgLeu2A-10 and ΔFgLeu2AB-8 could be completely or partially recovered when cultured in full-nutrition medium YEPD or PDA plates. The auxotroph caused by the disruption of *FgLEU2A* could also be overcome by exogenous addition of Leu into the culture medium. When cultured in FGA amended with Leu at 0.25 mM, the growth defects of the single deletion mutant ΔFgLeu2A-10 disappeared, while the concentration to rescue the auxotroph of the double deletion mutant ΔFgLeu2AB-8 should be higher than 0.25 mM (Fig 5).

### Auxotroph caused by *FgLEU2A* disruption is due to a block in the conversion of 3-IPPM to 2-KIC and Leu depletion in *F*. *graminearum*

**Fig 6:**
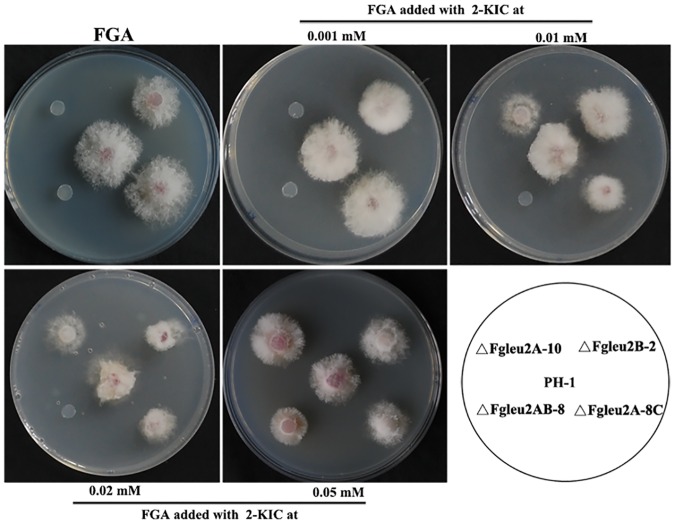
The auxotroph of the mutants can be satisfied by addition of the intermediate 2-KIC. Mycelial morphology of the wild-type PH-1, ΔFgLeu2A-10, ΔFgLeu2B-2, ΔFgLeu2AB-8 and ΔFgLeu2A-8C cultured on FGA medium amended with 2-KIC at different concentrations indicated in the figure at 25°C for 2 days.

As 2-KIC was the intermediate of the reaction catalyzed by enzyme IPMD encoded by *LEU2*, we further verify the functions of *FgLEU2A*and *FgLEU2B* by adding 2-KIC to FGA medium to test whether the mutants could be satisfied. As shown in Fig 6, the auxotroph ofΔFgLeu2A-10 and ΔFgLeu2AB-8 could be overcome by exogenous supplementation of 2-KIC. However, the minimum concentration to satisfy ΔFgLeu2A-10 or ΔFgLeu2AB-8 varied. ΔFgLeu2A-10, but not ΔFgLeu2AB-8 could survive when cultured in FGA amended with 2-KIC at 0.01 mM, the minimum concentration to rescue the auxotroph of ΔFgLeu2AB-8 should be higher than 0.05 mM.

### *FgLEU2A* is involved in the adaption to cellular stresses in *F*. *graminearum*

**Fig 7:**
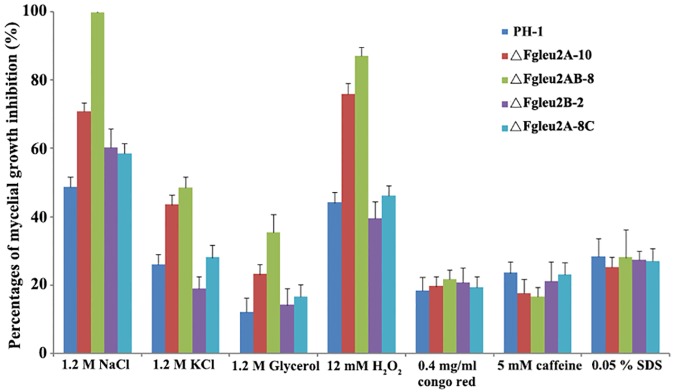
*FgLEU2A* is involved in adaptation to various cellular stresses in *F*. *graminearum*. Comparisons of mycelial inhibition percentages of each strain grown on YEPD medium amended with various cellular stresses at concentrations described in the figure and line bars in each column denote standard errors of three repeated experiments.

Results of the sensitivity assay showed that disruption of*FgLEU2A* caused reduced tolerance to osmotic and oxidative stresses, while deletion of *FgLEU2B* brought no significant changes to osmotic and oxidative stresses tested. As shown in Fig 7, ΔFgLeu2A-10 and ΔFgLeu2AB-8 showed significantly (*p*<0.05) increased sensitivity to all the four osmotic stresses mediated by NaCl, KCl, glycerol or D-srobitol at 1.2M as well as oxidative stresses mediated by H_2_O_2_ at 12 mM. The sensitivity of all the mutants to cell wall and cell membrane inhibitors did not change significantly as comparing to the wild-type strain PH-1.

### *FgLEU2A* plays a more important role in plant infection and DON accumulation in *F*. *graminearum*

**Fig 8:**
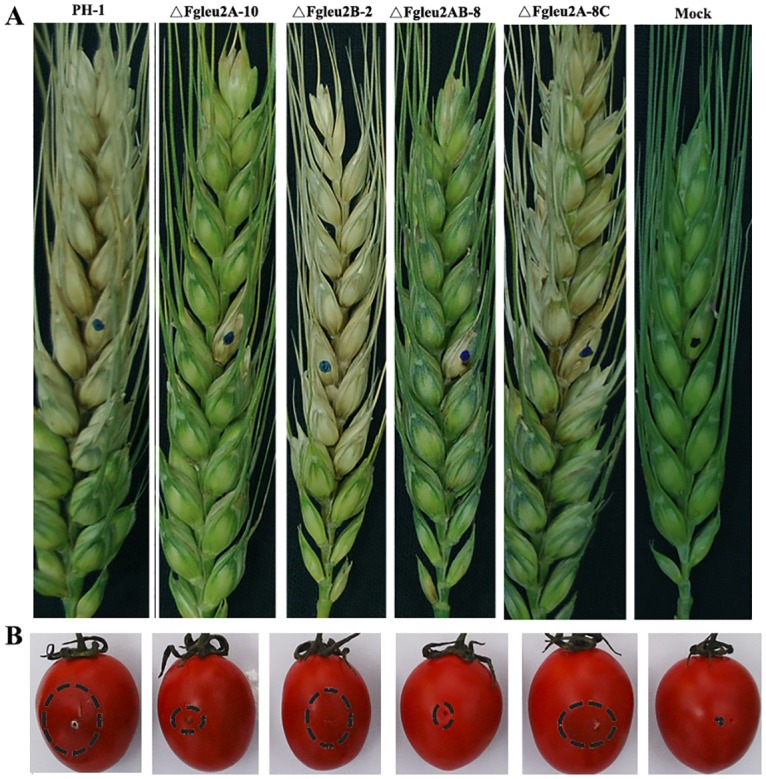
*FgLEU2A* is important for full virulence of *F*. *graminearum*. **A** Flowering wheat heads were point inoculated with a conidial suspension at 10^5^ conidia/ml of the wild type strain PH-1, ΔFgLeu2A-10, ΔFgLeu2B-2, ΔFgLeu2AB-8 and ΔFgLeu2A-8C and infected wheat heads were photographed 10 days after inoculation. **B** Cherry tomatoes were inoculated with a conidial suspension at 10^5^ conidia/ml of each strain and infected fruits were photographed 3 days after inoculation.

Virulence of *F*. *graminearum* strains was evaluated by point inoculating conidial suspension on flowering wheat heads and non-host cherry tomatoes. Results revealed the involvement of *FgLEU2A* but not *FgLEU2B* in full virulence. As shown in Fig 8A, ten days after inoculation, the wild type progenitor PH-1, ΔFgLeu2B-2, and ΔFgLeu2A-8C caused the typical scab symptoms in the inoculated as well as the nearby spikelets of flowering wheat heads. Under the same conditions, however, both *FgLEU2A*-deficent mutants lost aggressiveness in flowering wheat heads and the scab symptoms caused by ΔFgLeu2A-10 and ΔFgLeu2AB-8 were restricted in the inoculated spikelet. And for the non-host cherry tomatoes, the size of water-soaked rot lesion caused by ΔFgLeu2A-10 and ΔFgLeu2AB-8was significantly smaller as compared with the wild type progenitor PH-1, ΔFgLeu2B-2, and ΔFgLeu2A-8C (Fig 8B).

**Fig 9:**
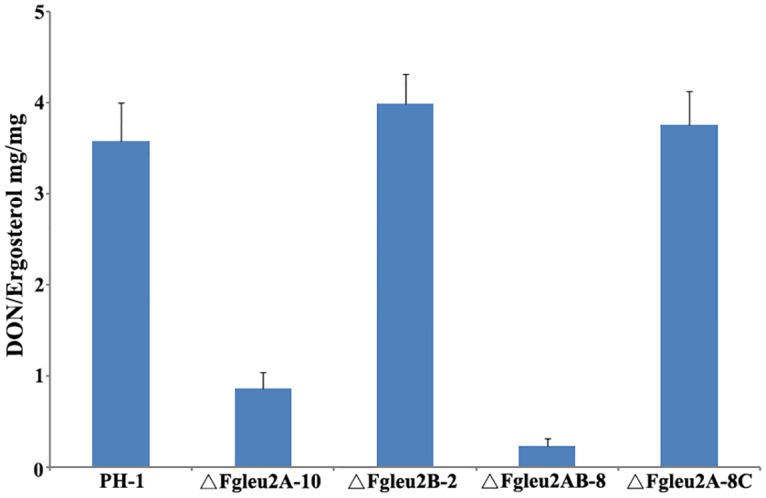
*FgLEU2A* is required for DON biosynthesis of *F*. *graminearum*. The amounts of DON (mg/mg ergosterol) produced by the wild type strain PH-1, ΔFgLeu2A-10, ΔFgLeu2B-2, ΔFgLeu2AB-8 and ΔFgLeu2A-8C in infected wheat kernels and bars denote standard errors from three repeated experiments.

The amount of DON accumulation was also compared in the wild-type and the mutants, and results showed that when cultured on sterilized wheat kernels for 20 days, the amounts of DON produced by the wild-type PH-1 or the complemented strain were 4 folds higher than that produced byΔFgLeu2A-10 and 16 folds higher than that produced by ΔFgLeu2AB-8 (significantly reduced, *p*<0.05) (Fig 9).

### Expression levels of *FgLEU2A/B* genes in the gene deletion mutants

**Fig 10:**
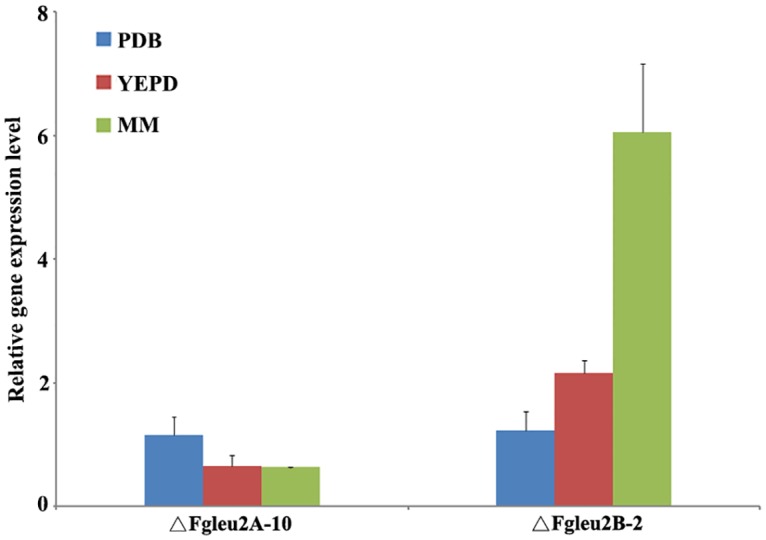
Relative expression of *FgLEU2A* and *FgLEU2B* in ΔFgLeu2A-10 and ΔFgLeu2B-2. The relative expression of *FgLEU2* genes is the relative amount of mRNA of each gene in the wild-type parent PH-1. Line bars in each column denote standard errors of three experiments.

Real-time PCR assays showed that the expression level of *FgLEU2A* in ΔFgLeu2B-2 was significantly (*p*< 0.05) higher than that in the wild-type parent PH-1 especially in the low-nutrition medium MM (fold change higher than 6.0) (Fig 10). Similar results were observed from other two *FgLEU2B* deletion mutants (data not shown). The expression levels of *FgLEU2B* in ΔFgLeu2A-10 did not change too much as compared with those in the wild-type parent PH-1 (Fig 10).

## Discussion

Yeast species including *Sacharomyces cerevisiae*, *Candia albicans* possess single *LEU2* gene encoding IPMD, the penultimate enzyme in the Leu biosynthesis. In this study, however, *LEU2* gene duplication was found in the genome of several filamentous fungi including *Aspergillus spp*., *Fusarium spp*., *Magnaporthe oryzae*, *Ustilago maydis* and *Sporisorium reilianum* (S1 Fig). In *F*. *graminaerum*, paralogous genes encoding one enzyme have been previously reported. We have characterized functions of three *cyp51* genes encoding 14-αdemethylases and two *ERG24* genes encoding sterol C-14 reductase of the ergosterol biosynthetic pathway, both enzymes are the targets of sterol biosynthesis inhibitors (SBIs). Targeted disruption of individual gene revealed different roles of these genes in mediating sensitivity to SBIs, however, deletion of single gene showed no effects on vegetative development or virulence [23,32]. In *A*. *fumigates*, Oliver *et al*., reported four putative *ILV3* (*ILV3A*-*D*) genes encoding DHAD with only Ilv3A required in BCAAs biosynthesis and full virulence [33]. Recently, Liu *et al*. characterized two Cdc2 kinases orthologs with distinct functions in vegetative and infectious hyphae in *F*. *graminaerum* [34]. When comparing the genome sequences of *S*. *cerevisiae* and *F*. *graminaerum*, we found 154 *F*. *graminearum* multiple genes (gene duplication numbers from 2 to 6) corresponding 63 *S*. *cerevisiae* single genes. Further investigation into the functions of these duplicated genes would underlie the evolutionary mechanism of the fungus.

Previous studies showed that *A*.*niger* elaborates two differentially expressed *LEU2* genes [8]. In *F*. *graminearum*, *FgLEU2A* and *FgLEU2B* were also differentially expressed in different cultures but the expression pattern of these two genes shares variations with *A*. *niger LEU2* genes. Expression of both *FgLEU2* genes could be detected during germination and hyphal formation phases and was greatly stimulated when cultured in low nutrition medium with *FgLEU2A* up-regulated in higher levels comparing with *FgLEU2B*. Different reactions of *FgLEU2A* and *FgLEU2B* to nutrition starvation at transcriptional level indicated a more crucial role of *FgLEU2A* in Leu biosynthesis in *F*. *graminearum*.

Functions of two *LEU2*orthologs have not been investigated in filamentous fungi. In this study, targeted disruption of individual *FgLEU2* gene in *F*. *graminearum* assigned different roles of FgLeu2A and FgLeu2B for Leu biosynthesis. The *FgLEU2A* deleted mutants, but not the *FgLEU2B* deletion mutant displayed Leu auxotroph. Exogenous supplementation of Leu or 2-KIC rescued the auxotroph of both ΔFgLeu2A-10 and ΔFgLeu2AB-8, indicating FgLeu2A, but not FgLeu2B, is dependent for Leu biosynthesis. Additionally, judged from the minimal concentration of exogenously-added Leu for satisfaction of different mutants, deletion mutants of *FgLEU2B* lacking Leu auxotroph does not make FgLeu2B absolutely useless in Leu biosynthesis. ΔFgLeu2AB-8 required a higher concentration Leu or 2-KIC from culture medium to overcome its auxotroph than ΔFgLeu2A-10, indicating a minor role of FgLeu2B in Leu biosynthesis in *F*. *graminearum*.

Apart from Leu biosynthesis, *FgLEU2A* and *FgLEU2B* also showed distinct roles in other cellular processes. Deletion mutants of *FgLEU2* showed different growth rates and aerial hyphal formation on different medium, indicating diverse roles of *FgLEU2A* and *FgLEU2B* in the utilizations of different nutrients. Significant down-regulation of red pigmentation genes in the *FgLEU2A* deleted mutants provided the molecular explanations for the reduced red pigment accumulation in these deletion mutants and assigned a more important role for *FgLEU2A* than *FgLEU2B* in secondary metabolism. *FgLEU2A* disruption mutants showed increased sensitivity to osmotic and oxidative stresses comparing with the wild type strain PH-1. The increased sensitivity to osmotic and oxidative stresses have also been observed in our previously characterized *ILV* mutants including *FgILV5*, *FgILV1*, *FgILV2* and *FgILV6* [19–21], and as the cellular stresses assay were conducted using YEPD medium in which all the mutants could acquire enough Leu from culture medium and show no obvious grown defects, which indicating the involvement of *FgLEU2* genes in the adaption to osmotic and oxidative stresses and this involvement is Leu-independent. Additionally, the inhibitory rate of ΔFgLeu2AB-8 was higher than that of ΔFgLeu2A-10, which is also in agreement with the different role weight of *FgLEU2A* and *FgLEU2B* in other cellular processes in *F*. *graminearum*.

Plant infection assays revealed reduced aggressiveness of *FgLEU2A* deleted mutants, which cannot be separated from the severe phenotypic defects exhibited by these mutants *in vitro*. The limited acquisition of Leu or 2-KIC from plant tissues the mutants encountered during colonization and mycelial spread within spikelets resulted in a series of infection-related defects including lower percentages of conidial germination, slower mycelial growth rate, reduced aerial hyphal formation, which may synthetically prevent the aggressiveness of the mutants within wheat heads. Second, it is well recognized that reactive oxygen species play crucial roles in host-pathogen interaction. When encountering pathogen attack, plants use the oxidative burst as an early defense reaction. *FgLEU2A*deficient mutants showed reduced tolerance to the oxidative stress mediated by H_2_O_2_, which may be related to the failed colonization or reduced aggressiveness in plant tissues. Additionally, DON, the end product of the trichothecene biosynthetic pathway, has been identified as an important virulence factor and plays a crucial role in the spread of the fungus through the infected wheat head, the decreased DON production caused by the disruption of *FgLEU2A* may also account for the reduced aggressiveness in ΔFgLeu2A-10 and ΔFgLeu2AB-8 [35–37].

Previous studies in *S*. *cerevisiae*, *C*. *albicans*, *Cryptococcus neoformans* and *Mycobacterium tuberculosis* indicated that certain genes encoding BCAA biosynthetic enzymes(e.g., *ILV2*) were important for fungal survive and/or virulence [13,15–17]. Recent reports in the rice blast fungus *M*. *oryzae* have revealed crucial roles of several BCAA biosynthetic genes in fungal development and full virulence in filamentous fungi [14,18].We recently identified several Val and Ile biosynthetic genes with distinct but crucial roles in mycelial and conidial morphogenesis, plant infection, and DON biosynthesis in the important wheat head scab fungus *F*.*graminearum* [19–21]. In this present study, a series of phenotypic defects displayed by the deletion mutants of *FgLEU2A* indicated the involvement of this Leu biosynthetic enzyme encoding gene in fungal development and infection in *F*. *graminearum*. The important functional roles of these BCAA biosynthetic genes, together with the fact that BCAA biosynthesis only exist in high plants, fungi and bacteria but not mammals, are making enzymes involved in BCAAs biosynthesis attractive and potential targets for the development of herbicides, fungicides and other antimicrobial compounds.

## Supporting Information

S1 Fig Phylogenetic tree generated by using the neighbor-joining method with Mega 5.10 software on the basis of deduced amino acid sequences. 1000 bootstrap replications and p-distance substitution model were used for the phylogeny test. The corresponding species name and protein IDs in FungiDB (http://fungidb.org) were labeled for each branch. Bootstrap values greater than 60 were displayed. Orange and green background colors indicate the defined group 1 and 2, respectively. Bold lines connect the two major clades (for *Aspergillus* and *Fusarium* genus, respectively) of Group 2 to the Group 1 clade corresponding to the same genus. Hollow stars indicate that there are multiple members from a species in Group 1. Black stars indicate FgLeu2a (FGSG_06675) and FgLeu2b (FGSG_17429), respectively.
(TIF)

S2 Fig Targeted disruption and complementation of *FgLEU2A*. **(A)** Gene replacement strategy for *FgLEU2A* gene. The hygromycin resistance cassette (*HPH*) is denoted by the large gray arrow. Primer binding sites are indicated by arrows (see S1 Table for the primer sequences). **(B)** Reverse transcription PCR analysis of *FgLEU2A* expression in PH-1, ΔFgLeu2A-10, ΔFgLeu2AB-8 and ΔFgLeu2A-8C using cDNA as template. NCK is a negative control without template cDNA in the PCR amplification. (**C**) Southern blot hybridization analysis of PH-1, ΔFgLeu2A-10, ΔFgLeu2AB-8 and ΔFgLeu2A-8C using a 941-bp *FgLEU2A* downstream fragment as a probe. Genomic DNA preparation of each strain was digested with *Nco* I.
(TIF)

S3 Fig Targeted disruption and complementation of *FgLEU2B*. **(A)** Gene replacement strategy for *FgLEU2B* gene. The hygromycin resistance cassette (*HPH*) is denoted by the large gray arrow. Primer binding sites are indicated by arrows (see S1 Table for the primer sequences). **(B)** Reverse transcription PCR analysis of *FgLEU2B* expression in PH-1 and ΔFgLeu2B-2 using cDNA as template. NCK is a negative control without template cDNA in the PCR amplification. (**C**) Southern blot hybridization analysis of PH-1 and ΔFgLeu2B-2 using a 954-bp *FgLEU2B* upstream fragment as a probe. Genomic DNA preparation of each strain was digested with *Nco* I.
(TIF)

S1 Table Oligonucleotide primers used in this study and their relevant characteristics. (DOCX)
