# Supplementary figures and images for: Two FgLEU2 Genes with Different Roles in Leucine Biosynthesis and Infection-Related Morphogenesis in Fusarium graminearum
Source: PLoS One. 2016 Nov 11;11(11):e0165927. doi: 10.1371/journal.pone.0165927 (PMC5106029; doi:10.1371/journal.pone.0165927)

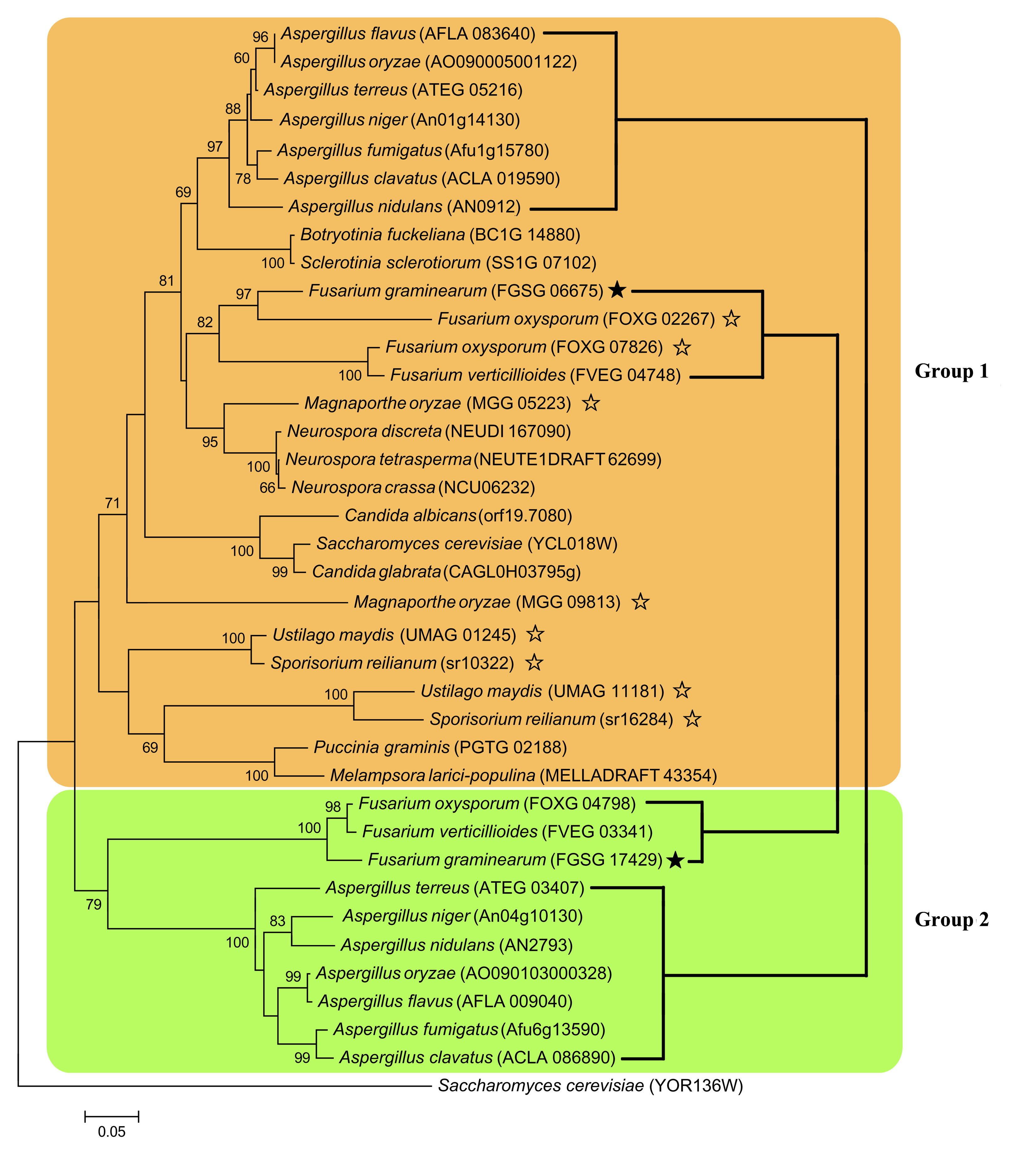

Supplement: S1 Fig — 1000 bootstrap replications and p-distance substitution model were used for the phylogeny test. The corresponding species name and protein IDs in FungiDB (http://fungidb.org) were labeled for each branch. Bootstrap values greater than 60 were displayed. Orange and green background colors indicate the defined group 1 and 2, respectively. Bold lines connect the two major clades (for Aspergillus and Fusarium genus, respectively) of Group 2 to the Group 1 clade corresponding to the same genus. Hollow stars indicate that there are multiple members from a species in Group 1. Black stars indicate FgLeu2a (FGSG_06675) and FgLeu2b (FGSG_17429), respectively. (TIF) [file pone.0165927.s001.tif]

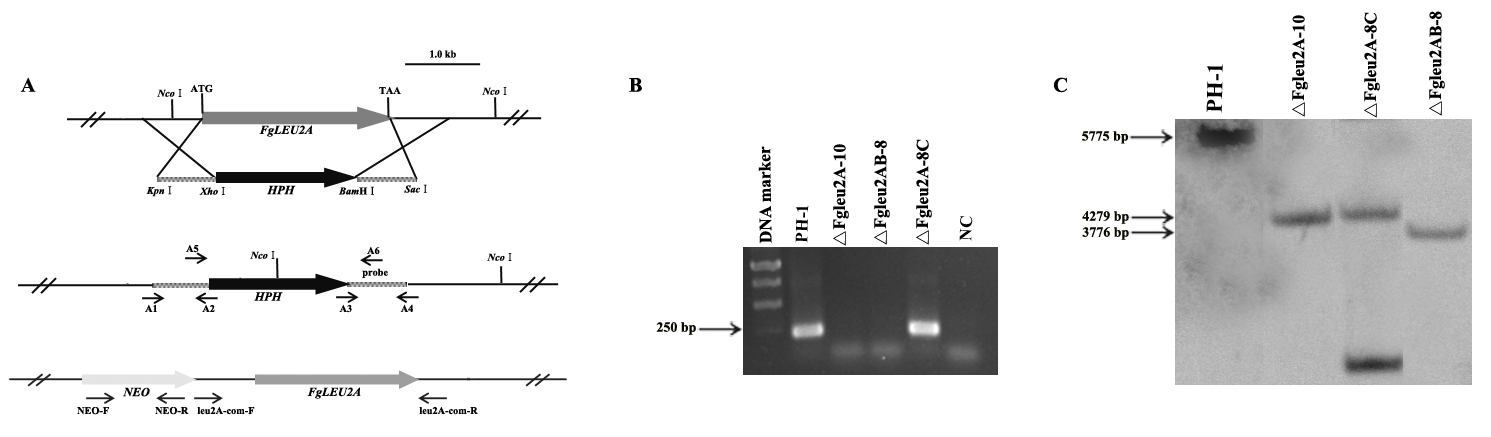

Supplement: S2 Fig — (A) Gene replacement strategy for FgLEU2A gene. The hygromycin resistance cassette (HPH) is denoted by the large gray arrow. Primer binding sites are indicated by arrows (see S1 Table for the primer sequences). (B) Reverse transcription PCR analysis of FgLEU2A expression in PH-1, ΔFgLeu2A-10, ΔFgLeu2AB-8 and ΔFgLeu2A-8C using cDNA as template. NCK is a negative control without template cDNA in the PCR amplification. (C) Southern blot hybridization analysis of PH-1, ΔFgLeu2A-10, ΔFgLeu2AB-8 and ΔFgLeu2A-8C using a 941-bp FgLEU2A downstream fragment as a probe. Genomic DNA preparation of each strain was digested with Nco I. (TIF) [file pone.0165927.s002.tif]

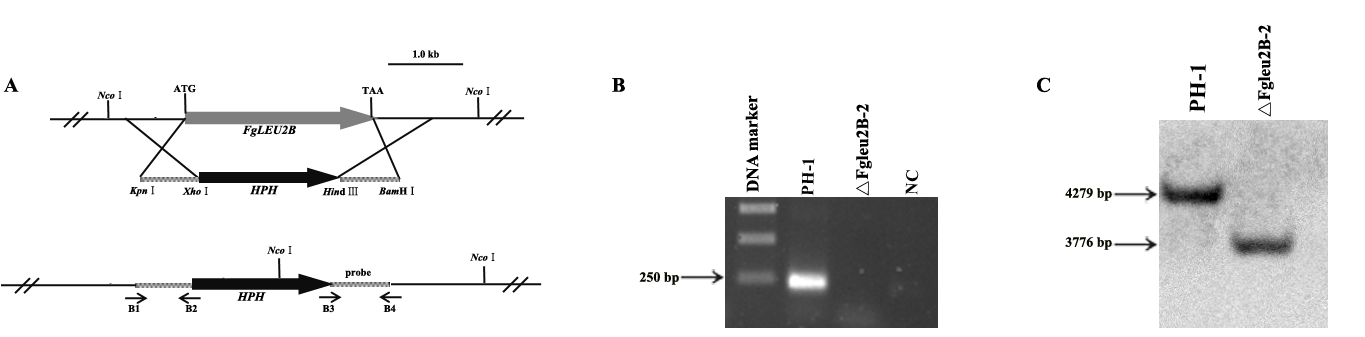

Supplement: S3 Fig — (A) Gene replacement strategy for FgLEU2B gene. The hygromycin resistance cassette (HPH) is denoted by the large gray arrow. Primer binding sites are indicated by arrows (see S1 Table for the primer sequences). (B) Reverse transcription PCR analysis of FgLEU2B expression in PH-1 and ΔFgLeu2B-2 using cDNA as template. NCK is a negative control without template cDNA in the PCR amplification. (C) Southern blot hybridization analysis of PH-1 and ΔFgLeu2B-2 using a 954-bp FgLEU2B upstream fragment as a probe. Genomic DNA preparation of each strain was digested with Nco I. (TIF) [file pone.0165927.s003.tif]
