# Supplementary material for: Two FgLEU2 Genes with Different Roles in Leucine Biosynthesis and Infection-Related Morphogenesis in Fusarium graminearum
Source: PLoS One. 2016 Nov 11;11(11):e0165927. doi: 10.1371/journal.pone.0165927 (PMC5106029; doi:10.1371/journal.pone.0165927)
Supplement: S1 Table — (DOCX) [file pone.0165927.s004.docx]

**S1 Table.** Oligonucleotide primers used in this study and their relevant characteristics.

| **Primer** | **Sequence(5’-3’)** | **Relevant characteristics** |
| --- | --- | --- |
| A1 | ATggtaccAATGGGACAAGGTGGGCAA | PCR primers for amplification of the upstream fragment of *FgLEU2A*. |
| A2 | ATctcgagAAAGTGTTGCACACGGCAT |  |
|  |  |  |
| A3 | ATggatccTGACGGCAGCTATATGAAGCT | PCR primers for amplification of the downstream fragment *of FgLEU2A.* |
| A4 | ATgagctcATGACTTTTCCCTCGCCTC |  |
|  |  |  |
| A5 | AGGACGGAGTTCTGGATGACA | PCR primers for the identification of *FgLEU2A* disruption mutants. |
| A6 | ACGGTTGCACTTGTAGGGAAA |  |
|  |  |  |
| B1 | ATggtaccTTGTACCATGTCAGCTCTGGC | PCR primers for amplification of the upstream fragment of *FgLEU2B*. |
| B2 | ATctcgagATTTCTGGGCCGACATGGTCT |  |
|  |  |  |
| B3 | ATaagcttTTAGCACTTATTGCGCGTTG | PCR primers for amplification of the downstream fragment *of FgLEU2B.* |
| B4 | ATggatccCTCAAATCCAGTTCATCGACA |  |
|  |  |  |
| B5 | TCAAACGTTCTTGACTACCGA | PCR primers for the identification of *FgLEU2B* disruption mutants. |
| B6 | GGAAAGGAGGCCTTGAAATT |  |
|  |  |  |
| leu2A-all-F | CCGATTCATTCAATCAATCAA | PCR primers for amplification of full cDNA sequence of *FgLEU2A.* |
| leu2A-all-F | TCCAATCCATCTAAGCCTTGA |  |
|  |  |  |
| leu2B-all-F | ATGTCGGCCCAGAAATTATGA | PCR primers for amplification of full cDNA sequence of *FgLEU2B.* |
| leu2B-all-F | CAACGCGCAATAAGTGCTAA |  |
|  |  |  |
| leu2A-com-F | ATggatccAATGGGACAAGGTGGGCAA | PCR primers for amplification of the entire *FgLEU2A* gene including 997-bp the promoter region and 1127-bp terminator region. |
| leu2A-com-R | ATtctagaATGACTTTTCCCTCGCCTC |  |
|  |  |  |
| leu2A-RT-F | TGACAAGAAAGTGTGGTCGCT | PCR primers for the detection of *FgLEU2A* transcription. |
| leu2A-RT-R | AGACCAATGCTTCCAGGGAT |  |
|  |  |  |
| leu2B-RT-F | CTCGACGTGATAGAAGCCAGT | PCR primers for the detection of *FgLEU2B* transcription. |
| leu2B-RT-R | AGACTCTGGTGTTGGCTCG |  |
|  |  |  |
| PKS12-RT-F | TGGTGTAGATGCTGTTCGTGT | PCR primers for analysis of *PKS12* expression. |
| PKS12-RT-R | TGAACTTTTCGAGGACGGAT |  |
|  |  |  |
| AurJ-RT-F | AAAAAGCAGCCAAGGAGCAT | PCR primers for analysis of *AurJ* expression. |
| AurJ-RT-R | TTCTGATGACACGCTCCCGTA |  |
|  |  |  |
| Gip1-RT-F | TGCGGTATCAGGTCACAAA | PCR primers for analysis of *Gip1* expression. |
| Gip1-RT-R | ATCAAAGTCTCCCACCGTGAA |  |
|  |  |  |
| Gip2-RT-F | CACCAGCCCTACACCATCTAA | PCR primers for analysis of *Gip2* expression. |
| Gip2-RT-R | TTTCCAAAGCGAGAAACAGC |  |
|  |  |  |
| AurF-RT-F | ATCTTCAGTCTTGACCATCCC | PCR primers for analysis of *AurF* expression. |
| AurF-RT-R | TACCCAAGATGTTCTGGCAA |  |
|  |  |  |
| Fgactin-F | ATCCACGTCACCACTTTCAA | PCR primers for amplification of the reference gene actin, in real-time PCR. |
| Fgactin-R | TGCTTGGAGATCCACATTTG |  |
|  |  |  |
| Neo-F | GGAGGTCAACACATCAATGCT | PCR primers for amplification of the G418 sulfate resistant gene *NEO*. |
| Neo-R | TCAGAAGAACTCGTCAAGAAG |  |
|  |  |  |
